# Metagenomic characterization of bacterial abundance and diversity in potato cyst nematode suppressive and conducive potato rhizosphere

**DOI:** 10.1371/journal.pone.0323382

**Published:** 2025-05-09

**Authors:** John Kamathi Kiige, Agnes Mumo Kavoo, Mwashasha Rashid Mwajita, Derleen Mogire, Stephen Ogada, Tofick Barasa Wekesa, Leonard Muriithi Kiirika

**Affiliations:** 1 Department of Agricultural Sciences, Karatina University, Karatina, Kenya; 2 Department of Horticulture and Food Security, Jomo Kenyatta University of Agriculture and Technology, Nairobi, Kenya; 3 Institute for Biotechnology Research, Jomo Kenyatta University of Agriculture and Technology, Nairobi, Kenya; 4 Novo Science Bio Solutions Limited, Nairobi, Kenya; University of Limpopo, SOUTH AFRICA

## Abstract

Potato (*Solanum tuberosum* L.) is an important food crop in Kenya, providing a source of nutrition and income for many farmers. However, potato cyst nematodes (PCN) cause significant damage to potato plants, leading to substantial economic losses and threatening the nation’s food security. Understanding the composition and functional potential of bacterial communities in the soil is important for developing sustainable biological control strategies against PCN and improving soil health. This cross-sectional purposive study examined the bacterial communities associated with PCN-suppressive and conducive potato rhizosphere from two major potato-producing counties in Kenya. We analyzed 180 soil samples from symptomatic and asymptomatic potato plants using shotgun metagenomics, followed by functional analysis to identify genes and metabolic pathways relevant to soil and plant health. Taxonomic classification revealed *Enterobacteriaceae* and *Pseudomonadaceae* as the most dominant bacterial families present. Within these families, the genera *Pseudomonas* and *Enterobacter* were highly abundant, both known for their plant growth-promoting traits, including biological control of soil pathogens and nutrient solubilization. KEGG and Pfam database analysis revealed pathways associated with nutrient cycling, transport systems, and metabolic functions. The abundance of iron-acquisition, chemotaxis, and diverse transport genes across analyzed samples suggests the presence of beneficial bacterial communities. This study provides the first report on bacterial ecology in PCN-infested rhizosphere in Kenya and its implications for soil health and PCN management.

## Introduction

Potato (*Solanum tuberosum* L.) is an important food crop consumed by over a billion people globally and supports the livelihoods of many farmers [1]. Kenya is the largest potato producer in East Africa and ranks third in Sub-Saharan Africa [2,3]. This crop contributes to food security and is a source of income for small-scale farmers in Kenya [4]. It generates approximately USD 500 million annually [3] and provides livelihoods for about 2.5 million Kenyans, including 800,000 farmers [5]. However, potato production in Kenya is threatened by pests and diseases. Among the most devastating pests is the potato cyst nematodes (PCN), *Globodera rostochiensis* Woll. [6,7]. This soil-borne pest causes significant yield losses [8] and is challenging to control due to its ability to persist in the soil for over 20 years [9,10]. Potato cyst nematodes attack the roots, hindering nutrient uptake and resulting in stunted growth, chlorosis, reduced tuber size, and total crop failure during severe infestations [11]. The management of PCN is complicated by the nematode’s life cycle and the difficulties associated with detecting and controlling them in the soil [10]. Traditional control methods, such as crop rotation, chemical nematicides, and resistant crop varieties, often fall short in many countries [12]. Therefore, there is a need to study microbial diversity and develop alternative sustainable methods of PCN control [3]. For instance, evaluating the rhizosphere as a potential source of natural PCN biocontrol agents is a promising area for research [13]. The rhizosphere is a habitat for a diverse microbial community, including bacteria that interact with plant roots [14]. Some microbial communities in the rhizosphere have been reported to suppress soil-borne pathogens, including nematodes, through competition, parasitism, and the production of inhibitory compounds [15]. According to [16], the continuous cropping in soils leads to the development of disease-suppressive soils. In contrast, conducive soils exhibit high disease severity when a pathogen, susceptible host, and favorable environment are present [17]. Monoculture of susceptible hosts aids in the evolution of suppressive soil [18]. Soils with the ability to suppress nematodes are potential sources of microorganisms that can be developed into biocontrol agents for plant nematodes [19]. One critical aspect of disease-suppression mechanisms is the composition of the microbial community [20]. Investigating the microbial composition of these suppressive soils can help identify specific bacterial species or consortia that play a role in PCN suppression [21].

Metagenomic analysis is a technique used to determine microbial diversity from environmental samples, providing a powerful tool for characterizing microbial communities in soils [21]. By sequencing the DNA of all microorganisms present in the soil, researchers can identify the diversity and abundance of bacterial species and understand their roles in either promoting or suppressing PCN populations [20]. This approach allows for a comprehensive analysis of the rhizosphere microbiome, revealing both known and previously unrecognized microbial interactions that could be harnessed for biological pest control [21].

Studies have identified bacterial genera, such as *Pseudomonas*, *Bacillus*, and *Streptomyces*, as potential biocontrol agents against plant pathogens [22]. These bacteria can produce antibiotics, lytic enzymes, and other bioactive compounds that directly impact pathogen survival or reproduction [22]. Moreover, some bacteria may induce systemic resistance in the host plant, enhancing its ability to withstand pathogen attacks [23]. However, the complexity of the rhizosphere microbiome means that many interactions in PCN-infested rhizosphere remain poorly understood, and metagenomic approaches are essential for uncovering the full spectrum of bacterial diversity and function. Identifying natural antagonistic bacteria from the PCN-suppressive potato rhizosphere and profiling their bioactive components can offer novel bio-nematicides against PCN [13].

This study utilized metagenomic techniques to characterize the bacterial communities associated with PCN-suppressive and conducive soils in potato fields in Nyandarua and Nyeri Counties, Kenya. By comparing the microbiome of the PCN-suppressive and conducive rhizospheres, the study aimed to identify specific bacterial taxa or functional genes that are present in PCN-symptomatic and asymptomatic potato rhizospheres. This knowledge could guide the development of novel biocontrol strategies, such as introducing beneficial bacteria into conducive soils or enhancing natural suppressiveness through soil management practices.

## Materials and methods

### Sampling design and sample collection

This study was done with approval by the National Commission for Science, Technology and Innovation (NACOSTI), Kenya, between January 2023 and August 2024 in Nyandarua (0°32’60.0“S, 36°36’60.0”E) and Nyeri (0.4167° S, 36.9500°E) to identify PCN-suppressive soils. These counties were selected based on their prominence in potato cultivation and documented cases of PCN infestation [7]. A stratified cross-sectional purposive approach was used during sample collection. To reduce sampling bias, the study areas were divided into sub-counties. Soil samples were collected from the rhizosphere of both symptomatic (conducive soils) and asymptomatic (suppressive soils) potato plants. 180 soil samples were collected from 90 potato farms randomly selected from potato-growing farmers’ groups and community-based organizations [7]. In each farm, two samples were collected: one from the rhizosphere of 10 potato plants exhibiting PCN infection symptoms as described by [24] and another from the rhizosphere of 10 potato plants showing no PCN infection symptoms. Samples were collected using a shovel at a depth of 20 cm, and each sample was thoroughly mixed in a bucket. The mixed samples were then packaged in sterile 200 g tubes and labeled.

The samples were transported in a cool box and stored at 4°C at Jomo Kenyatta University of Agriculture and Technology. Metadata for each farm was recorded, including GPS coordinates, land size, and agronomic practices (farming system, type of tillage, fertilizer or manure use, potato variety, and crop rotation).

### Sample preparation and pooling

The 180 collected samples were processed and pooled into four distinct groups based on their origin and the presence or absence of PCN symptoms. **S1:** Pool of 45 asymptomatic (suppressive) soil samples from Nyeri; **S2:** Pool of 45 symptomatic (conducive) soil samples from Nyeri; **S3:** Pool of 45 asymptomatic (suppressive) soil samples from Nyandarua; **S4:** Pool of 45 symptomatic (conducive) soil samples from Nyandarua.

Each pool (S1, S2, S3, and S4) was created by thoroughly mixing the corresponding individuals to ensure homogeneity. This pooling strategy was designed to minimize variability and enhance the representativeness of the samples for subsequent metagenomic analysis. After pooling, the soil samples were sieved to remove any large debris, such as stones and plant material, and homogenized further to create a uniform sample mixture. The homogenized samples were subdivided into aliquots for DNA extraction and other downstream analyses. All aliquots were stored at -20°C until further processing to preserve the integrity of the microbial DNA. This pooling approach was adopted to promote a comprehensive comparison of the microbial communities in suppressive versus conducive soils from different regions.

### DNA extraction

Genomic DNA was extracted from the soil using cetyl trimethyl ammonium bromide (CTAB) protocol, as described by [25]. Briefly, 5g of soil was ground into a fine powder using a mortar and pestle. Then, 600 μL of CTAB buffer (containing 0.2% β-mercaptoethanol) was added and incubated at 65°C for 30 min in a water bath. The homogenate was centrifuged at 16,000 g for 5 min. The supernatant was transferred to clean microcentrifuge tubes, and 25 μL of RNase A, 20 mg/mL lysozyme and 20 mg/mL proteinase K were then added and incubated at 37°C for 20 min. Following the incubation, equal volumes of phenol/chloroform/isoamyl alcohol (25:24:1) were added and centrifuged. The DNA was then precipitated using isopropanol and left overnight at 4°C. The DNA was pelleted by centrifugation, washed with 600 μL ice-cold 70% ethanol and dried at 25°C for 2 hours by inverting the tubes on a piece of tissue on a desk. The DNA was then suspended in 30 μL nuclease-free water and stored at −20°C. DNA samples were quantified using a Qubit 2.0 Fluorometer (Invitrogen).

### Sequencing and library preparation

The quality of the gDNA was determined using NanoDrop ™ 2000 spectrophotometer and Qubit fluorometer (Invitrogen, Thermo Fisher Scientific, Inc., Waltham, Massachusetts, USA), respectively according to manufacturer’s instructions. The gDNA was used to prepare libraries using TruSeq Sample Preparation Kits v2 according to the manufacturer’s instructions (Illumina, San Diego, CA, USA). Paired-end sequencing was then performed using the Illumina MiSeq platform (2 x 200 bp cycle) at Humanizing Genomics Macrogen, Netherlands, Europe.

### Metagenomic data processing and analysis

The quality of the paired-end raw sequence data was assessed using FastQC version 0.11.9 [26]. PRINSEQ version 0.20.4 [27] was used to filter out low-quality reads with a Phred quality score < 20 and short reads under 50 bp. The filtered reads were re-assessed using FastQC to ensure only high-quality reads were retained. A combined approach to metagenomics analysis was used to aim for higher resolution of microbial communities and improved functional annotation. MetaPhlAn version 4.0 [28] was used to analyze individual reads directly, while the SqueezeMeta pipeline version 1.6.3 [29] was employed to assemble reads into individual contigs first, followed by analysis.

The identification and quantification of the relative abundance of bacterial microbiota in each sample (taxonomic profiling) were performed using MetaPhlAn, with validation through the SqueezeMeta pipeline results. The filtered paired-end sequence reads in FASTQ format were mapped against a clade-specific marker gene reference database. The generated taxonomic assignment reports for each sample were merged for comparative and statistical analysis in R programming software. The relative abundances of bacterial genera and families within samples were visualized in bar plots and heatmaps using the *ggplot2* and *pheatmap* R packages in R version 4.3.2. Alpha diversity indices (Chao1, ACE, Shannon, Simpson, and Inverse Simpson) used to examine species richness, evenness and overall diversity were calculated using the R package *phyloseq*. Statistical differences in the alpha diversity indices were tested using the Wilcoxon rank-sum test. The bacterial diversity between counties and conditions (beta diversity) was analyzed and visualized through principal coordinate analysis (PCoA) using the *vegan* and *ggplot2* R packages, with Unweighted UniFrac, Bray-Curtis, and Jaccard distances. The statistical significances of the beta diversity analyses were estimated using a permutational multivariate analysis of variance (PERMANOVA) with 999 permutations.

SqueezeMeta, a pipeline encompassing all steps from assembly of reads to functional annotation, was run in the *coassembly* mode, where the reads from all samples were pooled, and a single assembly was performed. Then, reads from individual samples were mapped to the coassembly to obtain gene abundances in each sample. In the SqueezeMeta pipeline approach, assembly and taxonomic classification for comparison with MetaPhlAn results were done using Megahit [30] and the RDP classifier [31], respectively. Gene functional prediction was performed using Prodigal [32], and searches for homologous sequences against taxonomic and functional databases like BLAST non-redundant (nr), COG, and KEGG were performed using Diamond [33]. Searches for HMM homology were performed using HMMER3 [34] from the Pfam database [35].

## Results

### General overview of the sequence data

The number of reads obtained from each library is shown in Table 1.

**Table 1:** Raw sequenced data statistics.

Sample ID	Total bases(bp)	Total reads	Filtered reads	GC (%)	AT (%)	Q20 (%)	Q30 (%)
S1	2,075,701,568	13,746,368	13,651,222	55.6	44.4	98.0	94.0
S2	2,105,110,932	13,941,132	13,899,828	52.7	47.3	98.9	96.9
S3	2,115,589,728	14,010,528	13,978,966	55.8	44.2	98.7	96.5
S4	2,127,171,730	14,087,230	13,987,150	59.2	40.8	97.9	93.9

**Sample ID: Sample name; Total bases (bp): Total number of bases sequenced; Total reads: Total number of reads; ·GC (%): Ratio of GC content; AT (%): Ratio of AT content; Q20 (%): Ratio of bases that have a phred quality score of over 20; Q30 (%): Ratio of bases that have a phred quality score of over 30.**

**Fig 1:**
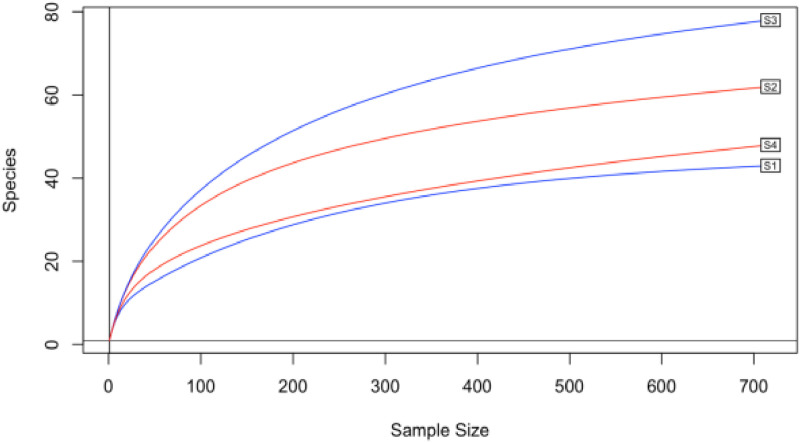
Rarefaction curves for samples clustered at a 90% sequence identity threshold. The chosen rarefaction depth is 90% of the dataset’s minimum sample depth. S1: Sample 1, S2: Sample 2, S3: Sample 3, and S4: Sample 4.

All of the samples approached a plateau in the rarefaction curves produced, indicating that the sample volumes were effective in assessing the taxonomic diversity of the soil samples (Fig 1).

### Taxonomic classification

**Fig 2:**
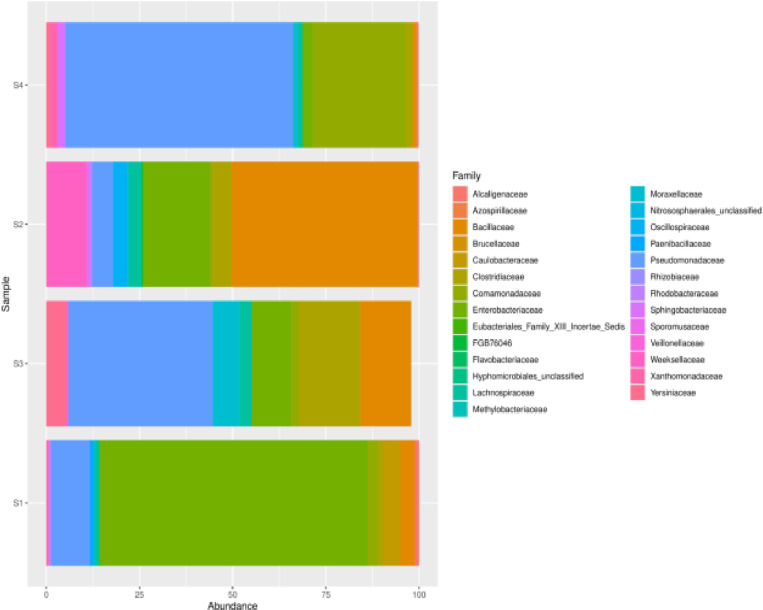
Bar chart showing bacterial family abundance in soil samples S1, S2, S3, and S4. A stacked column chart with taxonomic relative abundances (x-axis) by samples (y-axis). The height of each bar chart relates to the taxonomic relative abundance in a sample. For each soil sample type, S1: Sample 1, S2: Sample 2, S3: Sample 3, and S4: Sample 4.

Taxonomic family-level distributions for individual soil samples S1, S2, S3, and S4 are shown in Fig 2. In sample S1, the most abundant families were *Enterobacteriaceae* (72.10%) and *Pseudomonadaceae* (10.44%), with a notable presence of *Caulobacteraceae* (4.72%), *Bacillaceae* (3.83%), and *Comamonadaceae* (2.84%). Sample S2 was dominated by *Bacillaceae* (49.80%), *Enterobacteriaceae* (17.90%), and *Weeksellaceae* (10.31%), with significant counts of Pseudomonadaceae (5.65%) and *Clostridiaceae* (5.14%). Sample S3 showed high abundances of *Pseudomonadaceae* (38.72%), with considerable presence of *Clostridiaceae* (16.60%), *Bacillaceae* (13.69%) and *Enterobacteriaceae* (10.63%). Sample S4 had dominant families, including *Pseudomonadaceae* (61.10%) and *Comamonadaceae* (25.01%), with substantial counts of *Enterobacteriaceae* (2.71%). Other families, such as *Moraxellaceae*, *Yersiniaceae*, and *Lachnospiraceae,* were also detected across these samples, though in lower abundances.

**Fig 3:**
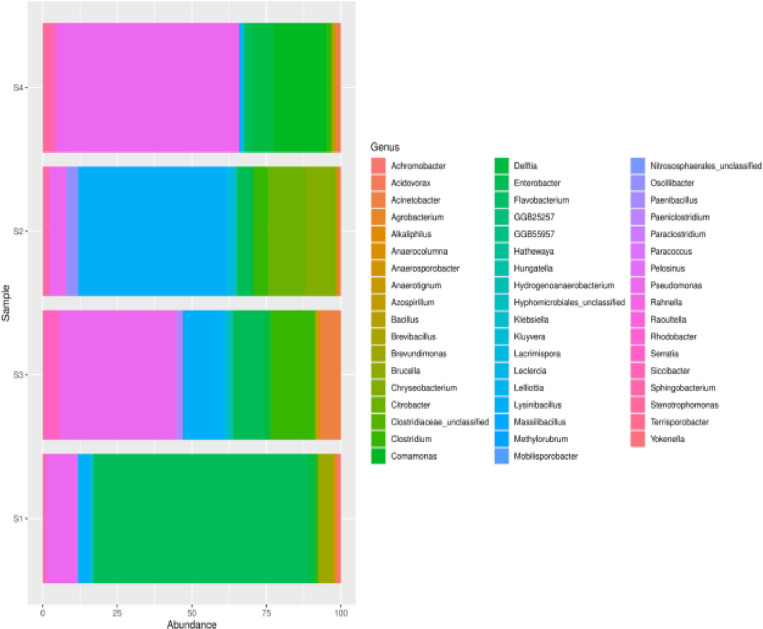
Bar chart showing bacterial genus abundance in soil samples S1, S2, S3, and S4. A stacked column chart with taxonomic relative abundances (x-axis) by samples (y-axis). The height of each bar chart relates to the taxonomic relative abundance in a sample. For each soil sample type, S1: Sample 1, S2: Sample 2, S3: Sample 3, and S4: Sample 4.

At the genus level, there was a significant diversity distribution of genera across the samples (Fig 3). For instance, in sample S1, the most abundant genera were *Enterobacter* (71.79%) and *Pseudomonas* (10.44%), with a notable presence of *Lysinibacillus* (3.83%) and *Brevundimonas* (4.72%). Sample S2 was dominated by *Lysinibacillus* (49.80%) with significant counts of *Chryseobacterium* (10.31%), *Citrobacter* (12.41%), *Pseudomonas* (5.65%), *Clostridium* (4.80%) and *Enterobacter* (4.71%) (Fig 3). Sample S3 showed high abundances of *Pseudomonas* (38.72%), with considerable presence of *Clostridium* (14.87%), *Lysinibacillus* (13.35%) and *Enterobacter* (10.48%), while Sample S4 had dominant genera of *Pseudomonas* (61.10%) with substantial counts of *Comamonas* (17.90%) and *Delftia* (7.10%). Other genera, such as *Chryseobacterium*, *Acinetobacter*, and *Stenotrophomonas,* were also detected across these samples, though in lower abundances (Fig 3).

**Fig 4:**
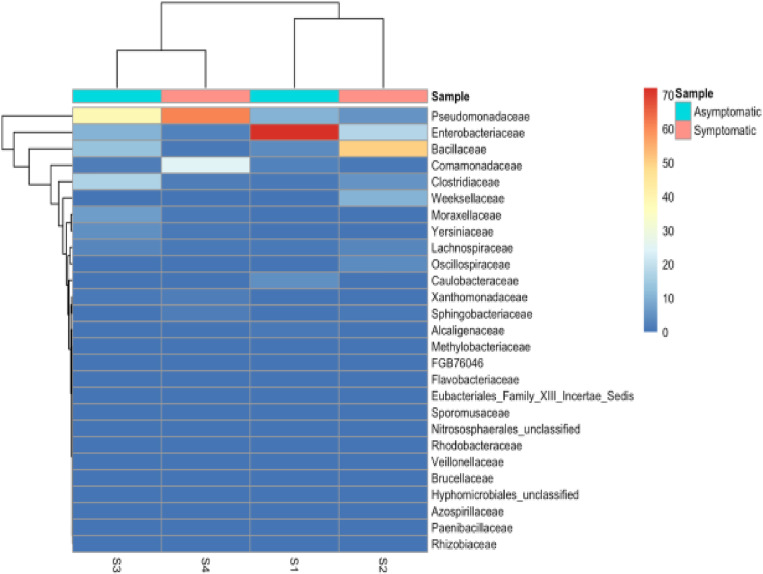
A heat map showing bacterial family abundance in Asymptomatic and symptomatic soil samples. The heat map represents log-transformed relative abundance values of bacterial genera across four soil samples, S1: Sample 1, S2: Sample 2, S3: Sample 3, and S4: Sample 4. (S1-S4). Rows represent different bacterial genera, while columns represent individual samples. The color scale ranges from blue (low abundance) to red (high abundance).

The hierarchical cluster map at the family level revealed two distinct groups of S1&S2 and S3&S4 (Fig 4). The results showed the dominance of *Pseudomonadaceae* and *Enterobacteriaceae* across all samples, with varying abundance levels between symptomatic and asymptomatic samples (Fig 4). *Bacillaceae* also shows notable presence across all samples, with higher abundance in S2 and S3. Other bacterial families, such as *Comamonadaceae, Clostridiaceae, Weeksellaceae, Moraxellaceae*, and *Yersiniaceae*, were also present across the samples but at lower abundances (Fig 4). The heat map also highlights differences between symptomatic and asymptomatic samples, with *Pseudomonadaceae* appearing to be most abundant in symptomatic samples (S2 and S4). At the same time, *Enterobacteriaceae* shows high abundance in asymptomatic samples (S1 and S3) (Fig 4).

**Fig 5:**
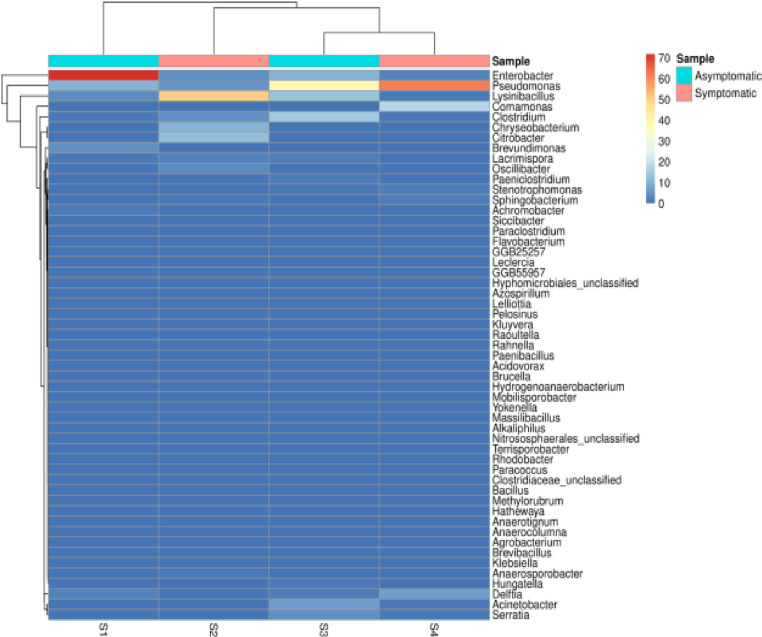
A heat map showing bacterial genus abundance in Asymptomatic and symptomatic soil samples. The heat map represents log-transformed relative abundance values of bacterial genera across four soil samples, S1: Sample 1, S2: Sample 2, S3: Sample 3, and S4: Sample 4. (S1-S4). Rows represent different bacterial genera, while columns represent individual samples. The color scale ranges from blue (low abundance) to red (high abundance).

The heat map illustrates the dominance of *Enterobacter* and *Pseudomonas* across all samples, displaying varying levels of abundance between symptomatic and asymptomatic samples (Fig 5). Other notable genera include *Lysinibacillus*, *Comamonas*, *Clostridium*, *Chryseobacterium*, *Citrobacter* and *Acinetobacter* (Fig 5). The heat map also highlights differences between symptomatic and asymptomatic samples. For instance, *Pseudomonas* appears to be more abundant in symptomatic samples, while *Enterobacter* shows high in asymptomatic samples (Fig 5).

### Alpha diversity

Diversity indices were calculated to assess the bacterial community diversity in each individual soil samples S1 to S4 (Table 2). The results show various diversity measures for each sample, including observed richness, Chao1, ACE, Shannon, Simpson, and Inverse Simpson indices.

**Table 2:** Standard diversity estimates for soil samples S1 to S4.

Sample	Observed	Chao1	ACE	Shannon	Simpson	Inverse Simpson
S1	43	45.33	47.11	2.728	0.9099	11.10
S2	62	72.11	73.83	3.3877378	0.9478	19.14
S3	78	88.93	91.31	3.52425059	0.9486	19.47
S4	48	65.14	68.93	2.94700814	0.9262	13.55

Based on observed richness, S3 had the highest number of observed species (78), followed by S2 (62), S4 (48), and S1 (43). This trend is consistent across the Chao1 and ACE estimators, which account for unobserved species. S3 showed the highest estimated richness (Chao1: 88.93, ACE: 91.31), while S1 had the lowest (Chao1: 45.33, ACE: 47.11). The Shannon diversity index, which considers both richness and evenness, was highest for S3 (3.52425059), followed closely by S2 (3.3877378). S1 and S4 had lower Shannon diversity values (2.728 and 2.94700814 respectively). This suggests that S3 and S2 have more diverse and evenly distributed bacterial communities compared to S1 and S4. The Simpson index, which gives more weight to dominant species, was highest for S1 (0.9099) and lowest for S4 (0.9262), though the differences were relatively small across all samples. The Inverse Simpson index, which increases with increasing diversity, was highest for S3 (19.47) and S2 (19.14), and lowest for S1 (11.10).

**Fig 6:**
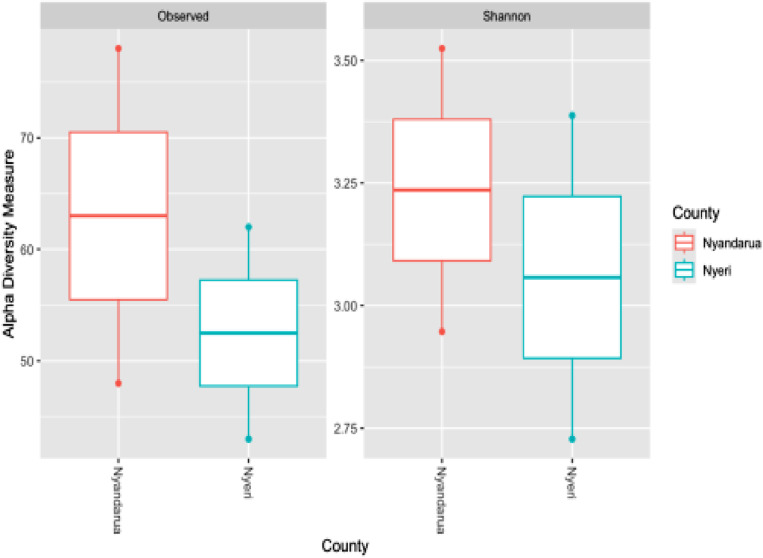
Alpha diversity measures by county, showing the Observed and the Shannon indices for the soil samples from Nyeri and Nyandarua counties.

Analysis of species richness (Observed number of OTUs) and community diversity (Shannon index) showed differences between the soil samples from two counties, Nyandarua and Nyeri (Fig 6). Nyandarua displayed a higher median value for the Observed OTU count than Nyeri, indicating greater bacterial species richness in Nyandarua. Additionally, Nyandarua exhibited a broader range of observed species, suggesting more variation in species richness across its samples. The Shannon index, which accounts for species richness and evenness, showed that Nyandarua also had a slightly higher median value than Nyeri, implying that Nyandarua had more bacterial species and a more balanced distribution of its species. However, the more extensive spread in the Shannon diversity values for Nyeri suggests greater variability in species evenness, meaning species distribution in Nyeri is less consistent across different samples.

**Fig 7:**
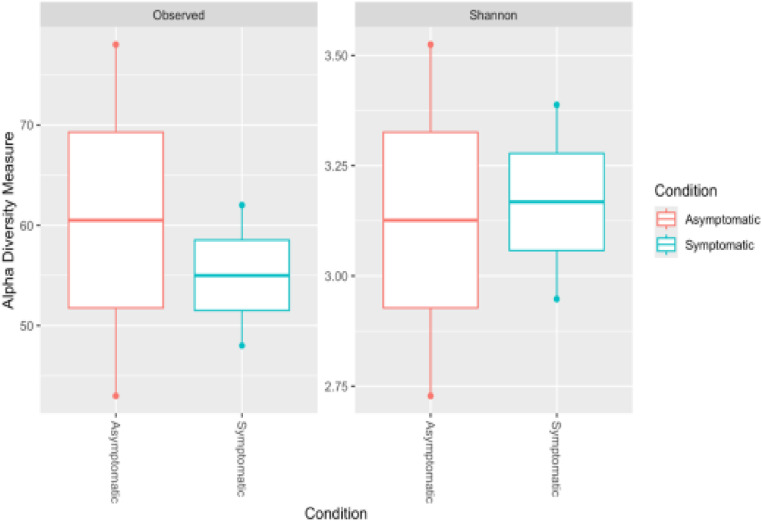
Alpha diversity measures by condition, showing the Observed and Shannon indices for samples from Asymptomatic and Symptomatic conditions.

The box plots (Fig 7) illustrate the alpha diversity measures, specifically Observed and Shannon indices, for Asymptomatic and Symptomatic conditions. The analysis revealed differences in alpha diversity between the two conditions. The Asymptomatic condition showed higher median values for both the Observed and Shannon indices than the Symptomatic condition, indicating greater species richness and a more even distribution of species in the asymptomatic samples. The range of values, as indicated by the whiskers, suggested variability in microbial diversity across the samples within each condition. Outliers in both conditions highlighted individual samples with particularly high or low diversity, pointing to localized variations in the microbial communities.

**Fig 8:**
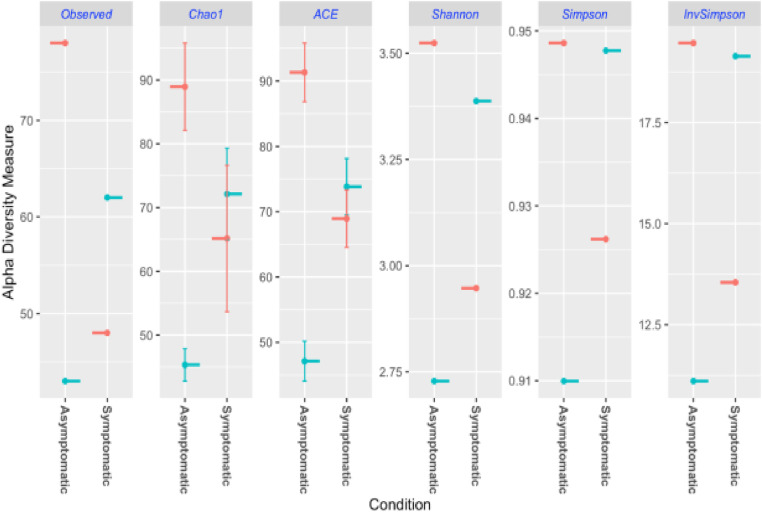
Alpha diversity estimates per condition, showing Observed, Chao1, ACE, Shannon, Simpson, and Inverse Simpson indices for Asymptomatic and Symptomatic samples.

Analysis of species richness (Observed number of OTUs) and community diversity (Chao1, ACE, Shannon, Simpson, and Inverse Simpson indices) showed that there were some differences between the two conditions, Asymptomatic and Symptomatic (Fig 8). The Observed, Chao1, and ACE indices, representing species richness, showed higher median values for the asymptomatic condition than the Symptomatic condition, suggesting greater bacterial species richness in asymptomatic samples. The Shannon index showed a slightly higher median value for the asymptomatic condition. The Simpson and Inverse Simpson indices, which emphasize dominant species, exhibited less pronounced differences between the two conditions. These results imply that there are some differences in bacterial community composition and diversity between asymptomatic and symptomatic samples, with asymptomatic samples generally showing more richness and diversity.

Pairwise comparisons using the Wilcoxon rank sum exact test were performed to determine whether the observed number of OTUs, Shannon diversity index, and Chao diversity index varied significantly between symptomatic and asymptomatic soil conditions, as well as between Nyeri and Nyandarua soil samples (Tables 3). For the comparison between symptomatic and asymptomatic soil conditions (Table 3), the test yielded a p-value of 1 for all three diversity measures (observed number of OTUs, Shannon diversity index, and Chao diversity index). This indicates no statistically significant differences in microbial community diversity between symptomatic and asymptomatic soil samples for any of the diversity indices evaluated. Additionally, for the comparison between Nyeri and Nyandarua soil samples (Table 3), the test yielded a p-value of 0.67 for all three diversity measures. This also signifies that there were no statistically significant differences in microbial community diversity between Nyeri and Nyandarua soil samples for any of the diversity indices assessed. These results imply that the microbial community diversity, measured by the observed number of OTUs, Shannon diversity index, and Chao diversity index, does not significantly differ between symptomatic and asymptomatic soil conditions or the two sampling locations (Nyeri and Nyandarua).

**Table 3:** Results of the Wilcoxon rank sum exact test comparing microbial community diversity across soil conditions and locations.

Comparison	Observed OTUs (p-value)	Shannon Index (p-value)	Chao Index (p-value)
**Symptomatic vs. Asymptomatic**	1.00	1.00	1.00
**Nyeri vs. Nyandarua**	0.67	0.67	0.67

### Beta diversity

**Fig 9:**
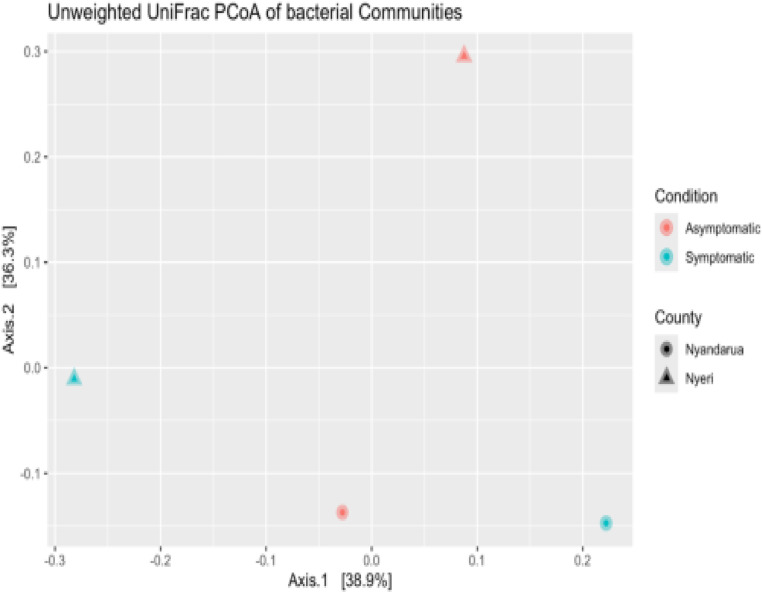
Unweighted UniFrac distance PCoA (Principal Coordinates Analysis) plot visualizing differences in bacterial communities from Nyeri and Nyandarua Counties.

**Fig 10:**
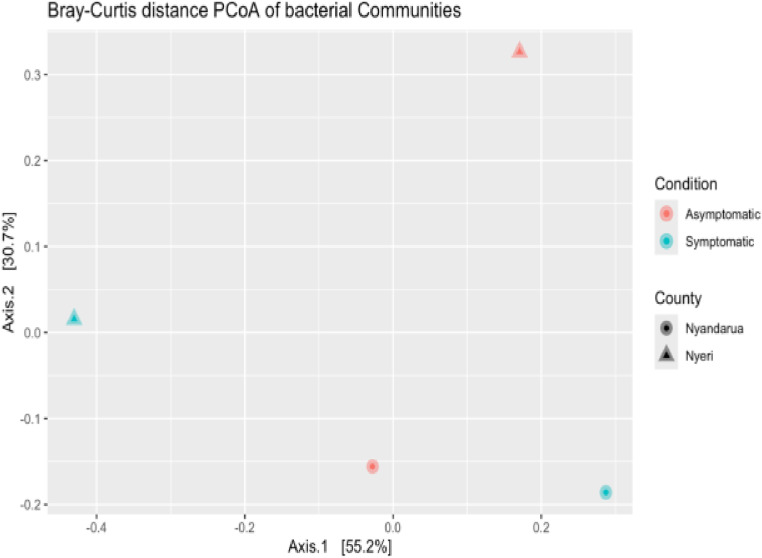
Bray-Curtis distance PCoA (Principal Coordinates Analysis) plot visualizing differences in bacterial communities from Nyeri and Nyandarua Counties.

**Fig 11:**
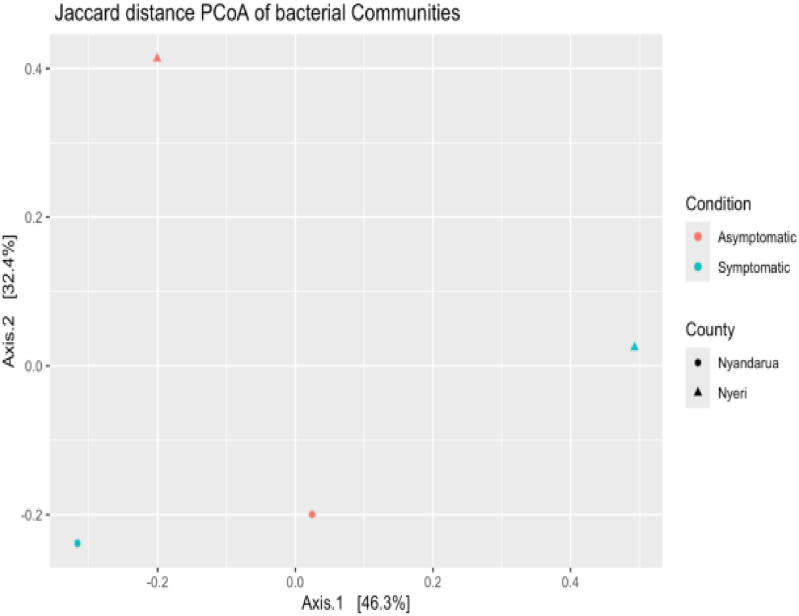
Jaccard-distance PCoA (Principal Coordinates Analysis) plot visualizing differences in bacterial communities from Nyeri and Nyandarua Counties.

Beta diversity analysis was performed to investigate the diversity between counties (Nyeri and Nyandarua) and conditions (Asymptomatic and Symptomatic). The Unweighted UniFrac, Bray-Curtis, and Jaccard distances were used for dimension reduction analysis (Figs 9–11). The PCoA plots show some separation between the two counties and between the two conditions. The Unweighted UniFrac PCoA plot demonstrates a distinct separation of bacterial communities in Nyeri and Nyandarua along the principal coordinate axis 1 (PC1), which accounts for 38.9% of the variation (Fig 9). A similar pattern is observed in the Bray-Curtis and Jaccard distance PCoA plots, with PC1 explaining 56.2% and 46.3% of the variation, respectively (Figs 10 and 11).

The separation was tested using the permutational analysis of variance (PERMANOVA), which examines whether the counties and conditions differ significantly. The PERMANOVA results for the Unweighted UniFrac distance show that the difference in diversity between conditions is not statistically significant (p > 0.05, P (>F) = 1), with approximately 27.40% of the variations being determined by the condition type (R² = 0.27) (Table 4). Similarly, the difference between the two counties is not statistically significant (p > 0.05, P (>F) = 0.67), with about 35.98% of the variations being determined by the county type (R² = 0.36) (Table 5).

**Table 4:** Permutational Multivariate Analysis of Variance (PERMANOVA) Unifrac distance for condition.

	Df	Sums Of Sqs	Mean Sqs	F.Model	R^2^	Pr(>F)
Condition	1	0.09654547	0.09654547	0.75477928	0.27398902	1
Residuals	2	0.25582439	0.12791219	NA	0.72601098	
Total	3	0.35236986			1	

Df: Degree freedom; Sqs: Square; R2: Linear Regression; Pr: Probability.

**Table 5:** Permutational Multivariate Analysis of Variance (PERMANOVA) Unifrac distance for county.

	Df	Sums Of Sqs	Mean Sqs	F.Model	R^2^	Pr(>F)
County	1	0.12676826	0.12676826	1.12382413	0.35975909	0.66666667
Residuals	2	0.2256016	0.1128008		0.64024091	
Total	3	0.35236986			1	

Df: Degree freedom; Sqs: Square; R2: Linear Regression; Pr: Probability.

The PERMANOVA results for the Bray-Curtis distance corroborate these findings, showing no statistically significant difference between conditions (p > 0.05, P (>F) = 1) (Table 6) or counties (p > 0.05, P (>F) = 0.67) (Table 7). The condition type accounts for about 20.04% of the variations (R² = 0.20) (Table 6), while the county type accounts for approximately 35.34% (R² = 0.35) (Table 7).

**Table 6:** Permutational Multivariate Analysis of Variance (PERMANOVA) Bray-curtis for conditions.

	Df	Sums of Sqs	Mean Sqs	F.Model	R^2^	Pr(>F)
Condition	1	0.10763	0.10763	0.50125	0.2004	1
Residuals	2	0.42943	0.21472		0.7996	
Total	3	0.53706			1.0000	

**Df: Degree freedom; Sqs: Square; R2: Linear Regression; Pr: Probability**

**Table 7:** Permutational Multivariate Analysis of Variance (PERMANOVA) Bray-Curtis distance for County.

	Df	Sums Of Sqs	MeanSqs	F.Model	R2	Pr(>F)
County	1	0.1898	0.1898	1.093	0.3534	0.6667
Residuals	2	0.3473	0.1736		0.6466	
Total	3	0.5371			1	

**Df: Degree freedom; Sqs: Square; R2: Linear Regression; Pr: Probability**

These results suggest that while there are observable differences in bacterial community composition between counties and conditions in the PCoA plots, these differences are not strong enough to be statistically significant. The analysis also indicates that geographical location (county) explains a larger proportion of the variation compared to health status (condition), although neither factor reaches statistical significance in this dataset.

### Functional annotation

**Fig 12:**
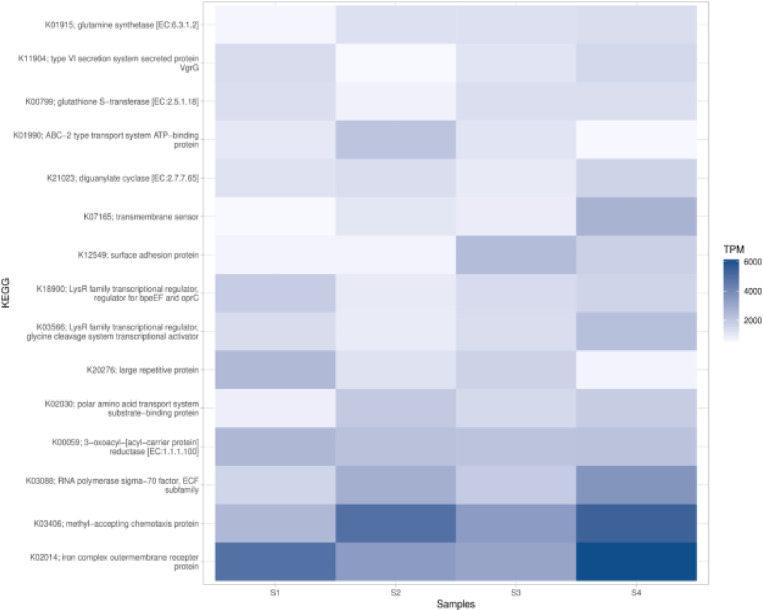
Heat map showing the abundance of the top functional genes detected in the soil samples from Nyeri and Nyandarua Counties.

The functional diversity of the microbial community in the soil samples from Nyeri and Nyandarua Counties was quantified by annotating metagenomic sequences with functions. Classification of assembled metagenomic protein sequences into protein families was performed by searching protein family databases. Protein-coding sequences were mapped against the Kyoto Encyclopedia of Genes and Genomes (KEGG) and Pfam databases. The relative abundance of functional genes was plotted as a heat map for each sample (Fig 12). The heat map analysis revealed variations in the abundance of different functional genes across the soil samples. Notably, the iron complex outer membrane receptor protein (K02014) and the methyl-accepting chemotaxis protein (KC3406) showed high abundance across all samples, particularly in S4. Glutamine synthetase (K01915) displayed varying levels of abundance among the samples. Some genes, such as the type VI secretion system secreted protein VgrG (K11904), showed consistently low abundance across all samples (Fig 12).

**Fig 13:**
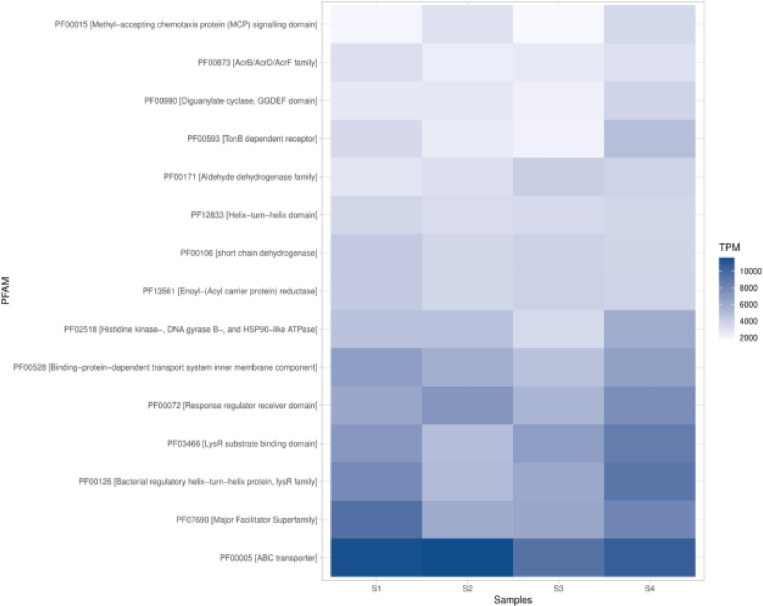
Heat map showing the abundance of the top protein families detected in the soil samples from Nyeri and Nyandarua Counties. **(TPM)**-**Transcripts Per Million.**

Additionally, the Pfam analysis revealed that ABC transporter (PF00005) and Major Facilitator Superfamily (PF07690) were highly abundant across all soil samples (Fig 13). The bacterial regulatory helix-turn-helix protein, lysR family (PF00126), showed varying levels of abundance, with higher levels in S1 and S4. Environmental information processing functions, represented by proteins such as the Methyl-accepting chemotaxis protein (MCP) signaling domain (PF00015), were detected but at lower abundance. The analysis also showed that protein families related to cellular processes and metabolic functions were present in varying abundances across the samples. For instance, the Short-chain dehydrogenase (PF00106) and Acyl-CoA dehydrogenase family (PF02771) showed moderate abundance, indicating the presence of metabolic activities in the soil microbial communities (Fig 13).

## Discussion

Several studies have emphasized the role of soil microbiome in plant health and agricultural productivity. This study aimed to investigate the soil microbial communities in potato fields located in Nyeri and Nyandarua counties, focusing on their potential roles in potato cyst nematode (PCN) suppression and improving overall soil health. It is essential to evaluate the community profiles of microorganisms present in the soil, particularly in regions where crop diseases are common. In recent years, studies using shotgun metagenomic sequencing technologies have become increasingly popular for examining microbial populations in soil ecosystems [36,37]. The rarefaction curve analysis of our samples shows that all samples approached a plateau, suggesting that the sample volumes were effective in estimating soil microbial taxa. This indicates that the sampling depth was sufficient to capture the majority of microbial diversity present in these soils, providing a reliable basis for downstream taxonomic and functional analyses. This is consistent with observations by other researchers in soil microbiome studies [38]. The taxonomic classification at both family and genus levels revealed a complex microbial community structure in the soil samples from Nyeri and Nyandarua Counties.

At the family level, *Enterobacteriaceae* and *Pseudomonadaceae* were found to be dominant across all samples, with varying levels of abundance between symptomatic and asymptomatic samples. This is particularly interesting as members of these families are known to include both plant pathogens and beneficial bacteria [39,40]. The dominance of these families suggests their potential involvement in plant health and disease processes, emphasizing the dual role these bacteria may play depending on environmental conditions and microbial interactions. The high abundance of *Bacillaceae* across all samples, particularly in S2 and S3, could indicate their important role in soil health, as many Bacillus species are known to promote plant growth and suppress plant pathogens [41,42].

At the genus level, *Enterobacter* and *Pseudomonas* were the most abundant across all samples. The higher abundance of *Pseudomonas* in symptomatic samples (S2 and S4) is noteworthy, as some *Pseudomonas* species are known plant pathogens, while others can be beneficial to plants [43,44]. The finding aligns with the complex role of *Pseudomonas* in the rhizosphere, where certain strains can act as biocontrol agents by producing antimicrobial compounds, while others may contribute to disease progression [22]. The hierarchical clustering analysis revealed similarities in bacterial community composition within sample pairs, which could be related to geographical proximity or similar environmental conditions. This consistent clustering at both family and genus levels highlights the stability and reliability of the observed community structure. The differences observed between symptomatic and asymptomatic samples provide valuable insights into potential associations between specific bacterial taxa and plant health in these regions. The clustering of symptomatic samples (S2 and S4) with high abundances of *Pseudomonas* suggests a potential link between these bacterial communities and disease conditions, supporting the hypothesis that microbial community composition can influence soil suppressiveness against pathogens [21]. The elevated prevalence of *Pseudomonas* in symptomatic samples suggests its potential role in plant disease progression. However, it’s crucial to note that further research is needed to establish causal relationships. Additionally, the presence of beneficial *Pseudomonas* strains in disease-suppressive soils highlight the need to differentiate between pathogenic and protective strains within this genus [45].

The analysis of soil microbiome in Nyeri and Nyandarua Counties provides valuable insights into the bacterial community structure and diversity in these agricultural regions. Understanding these microbial communities is crucial, as soil microbiome play a significant role in plant health, nutrient cycling, and overall ecosystem functioning [46–49]. The alpha diversity metrics, including the observed OTUs and Shannon index, indicate that asymptomatic soils generally exhibit higher microbial diversity and richness than symptomatic soils. This finding supports the hypothesis that diverse microbial communities are more effective in suppressing soil-borne pathogens, as a higher diversity of microbes can occupy ecological niches and outcompete pathogenic organisms [21]. The higher richness and evenness in asymptomatic soils may thus contribute to their suppressive capabilities against PCN.

The comparison between symptomatic and asymptomatic conditions showed higher alpha diversity in asymptomatic samples across multiple indices (Observed, Chao1, ACE, and Shannon). This could suggest that a more diverse microbial community might be associated with healthier soil conditions, aligning with the concept of microbial diversity contributing to ecosystem resilience [50,51]. However, it’s important to note that the Wilcoxon rank sum exact tests did not find these differences statistically significant, highlighting the complex nature of soil microbial communities and the need for larger sample sizes in future studies.

Beta diversity analysis using Unweighted UniFrac, Bray-Curtis, and Jaccard distances revealed some separation between counties and conditions in the PCoA plots. The PERMANOVA results, however, showed that these differences were not statistically significant. Despite observable trends, this lack of statistical significance in alpha and beta diversity analyses between counties and conditions emphasizes the need to account for biological and environmental factors that may shape microbial community composition. This indicates that more nuanced approaches or larger datasets might be necessary to fully capture these effects [52,53]. This suggests that while there are observable trends in community composition between counties and conditions, other factors may strongly influence microbial community structure [54,55]. This similarity across different soils could be attributed to shared agricultural practices or similar environmental pressures that consistently shape microbial communities, regardless of the symptomatic status of the plants or geographical differences.

Our analysis of individual samples (S1-S4) showed notable variations in diversity indices. Sample S3 consistently displayed the highest diversity across multiple indices, while S1 generally showed the lowest. These differences could be related to specific soil properties, plant species present, or other environmental factors at each sampling site [56–58]. The higher diversity observed in S3 might indicate a more complex and potentially more resilient microbial community, which could be beneficial for plant health and soil stability. In contrast, the lower diversity in S1 suggests a more specialized or less resilient community, which might be more susceptible to environmental changes or pathogen invasion [58,59].

The functional annotation of soil microbiomes from Nyeri and Nyandarua Counties provides valuable insights into the metabolic capabilities and potential ecological roles of these microbial communities. By linking taxonomic data with functional gene profiles, this study offers a more comprehensive understanding of the microbial processes that underpin soil health in these regions. This analysis complements the taxonomic profiling by elucidating the functional diversity present in these agricultural soils.

Our analysis using the Kyoto Encyclopedia of Genes and Genomes (KEGG) database revealed variations in the abundance of different functional genes across the soil samples. The high abundance of iron complex outer membrane receptor protein (K02014) and methyl-accepting chemotaxis protein (KC3406) across all samples, particularly in S4, suggests the importance of iron acquisition and chemotaxis in these soil microbial communities [60,61]. These functions are crucial for bacterial survival and adaptation in soil environments, where nutrient availability and chemical gradients play significant roles in microbial ecology [60]. Iron acquisition and chemotaxis are essential processes for microbial colonization and survival in competitive soil environments, which could influence the overall microbial community structure and function.

The varying levels of glutamine synthetase (K01915) abundance among the samples indicate differences in nitrogen metabolism, a critical process in soil nutrient cycling and plant-microbe interactions [62,63]. The consistently low abundance of the type VI secretion system secreted protein VgrG (K11904), particularly in S2, suggests that this bacterial competition mechanism may be less prevalent in these soil communities [64,65]. These findings highlight the potential importance of nitrogen metabolism in shaping soil microbial communities, particularly in agricultural contexts where nitrogen availability can be a limiting factor for both plant and microbial growth.

Complementing the KEGG database analysis, additional analysis using the Pfam database provided further insights into the protein family composition of these soil microbiomes. The high abundance of ABC transporter (PF00005) and Major Facilitator Superfamily (PF07690) across all soil samples underscores the importance of diverse transport systems in these microbial communities. These transport proteins play crucial roles in nutrient uptake, xenobiotic efflux, and cell-to-cell communication, which are essential for microbial survival and interactions in soil ecosystems [66,67]. The prominence of these transport systems suggests that nutrient acquisition and stress responses are key functions in these soil microbiomes, which could be essential for maintaining soil health and fertility in agricultural settings.

The varying levels of bacterial regulatory helix-turn-helix protein, lysR family (PF00126), with higher levels in S1 and S4, suggest differences in transcriptional regulation among the samples. This could indicate varying adaptive responses to environmental conditions between the sampling sites [68,69]. Differences in regulatory protein levels may reflect distinct environmental pressures or resource availability at each site, which could influence microbial community dynamics and functional capabilities. The presence of protein families related to cellular processes and metabolic functions, such as Short-chain dehydrogenase (PF00106) and Acyl-CoA dehydrogenase family (PF02771), at moderate abundances indicates active metabolic processes in these soil microbial communities. These enzymes are involved in various metabolic pathways, including lipid metabolism and xenobiotic degradation, which are essential for soil health and potential bioremediation applications [70,71]. The metabolic diversity observed in these samples suggests that these microbial communities are capable of a wide range of biochemical processes, which could be harnessed for agricultural and environmental applications.

The observed functional diversity and varying patterns of gene and protein family abundance between samples suggest that the microbial communities in different soil samples from Nyeri and Nyandarua Counties have distinct functional profiles. These differences could be driven by various factors such as soil physicochemical properties, plant species present, or land management practices [72–75]. Understanding these functional profiles could provide valuable insights into how soil management practices influence microbial community functions and, consequently, soil health and crop productivity.

This functional annotation provides a deeper understanding of the potential metabolic capabilities and ecological roles of the soil microbiome in these agricultural regions. By linking these functional capabilities with specific microbial taxa, this study offers a foundation for developing targeted strategies to enhance soil health and crop productivity. The insights gained from this analysis could have important implications for soil health management and agricultural productivity. For instance, the abundance of genes related to nutrient cycling and transport systems could inform strategies for optimizing fertilizer use and enhancing nutrient uptake efficiency in crops [76,77].

Future research could focus on linking these functional profiles with specific soil properties and plant health indicators to develop targeted approaches for improving soil fertility and crop productivity in these regions. Furthermore, there is a need for future studies with larger sample sizes in order to establish more reliable causal relationships in PCN suppressive soils. Additionally, investigating the temporal dynamics of these functional profiles across different seasons or under various agricultural practices could provide valuable information for sustainable soil management strategies [78–80].

## Conclusions

This study provides a metagenomic characterization of bacterial communities in potato rhizosphere under potato cyst nematode suppressive and conducive soils in Nyandarua and Nyeri counties, Kenya. The findings show that *Enterobacteriaceae* and *Pseudomonadaceae* were the dominant bacterial families in the rhizosphere, with observed differences in their abundance between symptomatic and asymptomatic soil samples. Additionally, functional metagenomic analysis identified key microbial processes, including nutrient cycling, iron acquisition, chemotaxis, and transport mechanisms, which are essential for microbial survival and adaptation in soil environments. The presence of beneficial bacterial species with potential antagonistic properties against PCN suggests the possibility of utilizing microbial-based strategies for sustainable nematode management.

The study also reveals higher diversity and richness in asymptomatic soils which suggest that microbial community composition might have a role in determining soil suppressiveness against potato cyst nematode. Although no statistically significant differences were found between symptomatic and asymptomatic soils at the alpha and beta diversity levels, the observed trends indicate that further research with larger sample sizes and seasonally replicated studies could provide deeper insights into microbial interactions and their impact on soil suppressiveness.

Future research should focus on isolating and characterizing specific bacterial strains from suppressive soils to evaluate their efficacy as biocontrol agents. Greenhouse and field experiments should be conducted to determine how these bacteria interact with PCN populations and their influence on potato plant growth and yield.

Furthermore, comparative studies across different potato-growing regions in Kenya and beyond will help determine whether the microbial trends observed in this study are widespread or region-specific. Ultimately, this study provides a foundation for future research aimed at harnessing beneficial soil microbes for enhanced potato productivity and soil health management.
