# Correction: Metagenomic characterization of bacterial abundance and diversity in potato cyst nematode suppressive and conducive potato rhizosphere

**DOI:** 10.1371/journal.pone.0342019

**Published:** 2026-01-29

**Authors:** John Kamathi Kiige, Agnes Mumo Kavoo, Mwashasha Rashid Mwajita, Derleen Mogire, Stephen Ogada, Tofick Barasa Wekesa, Leonard Muriithi Kiirika

The reference list is incorrectly numbered. The correct list is:

## References

Food and Agriculture Organization of the United Nations (FAO). FAOSTAT. Rome: FAO; 2021. Available from: https://www.fao.org/faostat/en/Gildemacher PR, Kaguongo W, Ortiz O, Tesfaye A, Woldegiorgis G, Wagoire WW, et al. Improving potato production in Kenya, Uganda and Ethiopia: A system diagnosis. Potato Res. 2009;52:173-205. https://doi.org/10.1007/s11540-009-9127-4Nyang'au MN, Akutse KS, Fathiya K, Charimbu MK, Haukeland S. Biodiversity and efficacy of fungal isolates associated with Kenyan populations of potato cyst nematode (Globodera spp.). Biol Control. 2023;186:105328. https://doi.org/10.1016/j.biocontrol.2023.105328International Potato Center (CIP). Promoting nutrition-sensitive potato value chains in Kenya [Internet]. Lima, Peru: CIP; [cited 2025 Mar 3]. Available from: https://cipotato.org/cip_projects/promoting-nutrition-sensitive-potato-value-chains-kenyaJanssens SRM, Wiersema SG, Goos HT. The value chain for seed and ware potatoes in Kenya: Opportunities for development (No. 13-080). LEI Wageningen UR; 2013. https://edepot.wur.nl/279729Trudgill DL, Blok VC. Apomictic, polyphagous root-knot nematodes: Exceptionally successful and damaging biotrophic root pathogens. Annu Rev Phytopathol. 2001; 39:53-77. https://doi.org/10.1146/annurev.phyto.39.1.53, PMid:11701859Mburu H, Cortada L, Haukeland S, Ronno W, Nyongesa M, Kinyua Z, et al. Potato cyst nematodes: A new threat to potato production in East Africa. Front Plant Sci. 2020; 11:670. https://doi.org/10.3389/fpls.2020.00670, PMid:32523602 PMCid:PMC7261874Niere B, Karuri H. Nematode parasites of potato and sweet potato. In: Plant parasitic nematodes in subtropical and tropical agriculture. 2nd ed. 2018;222-51. https://doi.org/10.1079/9781786391247.0222Jones JT, Haegeman A, Danchin EGJ, Gaur HS, Helder J, Jones MGK, et al. Top 10 plant-parasitic nematodes in molecular plant pathology. Mol Plant Pathol. 2013; 14(9):946-61. https://doi.org/10.1111/mpp.12057, PMid:23809086 PMCid:PMC6638764Fatemy S, Ahmarimoghadam P. The role of some agricultural crops and weeds on decline of potato cyst nematode Globodera rostochiensis and their possible use as trap crops. J Crop Prot. 2019;8(2):191-200. 20.1001.1.22519041.2019.8.2.9.1Ali M, Azeem F, Abbas A, Joyia F, Li H, Dababat A. Transgenic strategies for enhancement of nematode resistance in plants. Front Plant Sci. 2017;8(750):1-13. https://doi.org/10.3389/fpls.2017.00750, PMid:28536595 PMCid:PMC5422515Oerke EC. Crop losses to pests. J Agric Sci. 2006;144(1):31-43. https://doi.org/10.1017/S0021859605005708Bairwa A, Venkatasalam EP, Priyank HM, Sharma S. Introduction of potato cyst nematodes, life cycle and their management through biobased amendments. In: Microbial biotechnology in crop protection. Singapore: Springer Singapore; 2021;79-95. Available from: https://www.researchgate.net/publication/352055211 [accessed Aug 19 2024].Berendsen RL, Pieterse CMJ, Bakker PAHM. The rhizosphere microbiome and plant health. Trends Plant Sci. 2012;17(8):478-86. https://doi.org/10.1016/j.tplants.2012.04.001Mazzola M. Mechanisms of natural soil suppressiveness to soilborne diseases. Antonie Van Leeuwenhoek. 2002;81(1-4):557-64. https://doi.org/10.1023/A:1020557523557Raaijmakers JM, Mazzola M. Soil immune responses. Science. 2016;352(6292):1392-3. https://doi.org/10.1126/science.aaf3252, PMid:27313024Kariuki GM, Muriuki LK, Kibiro EM. The impact of suppressive soils on plant pathogens and agricultural productivity. In: Organic amendments and soil suppressiveness in plant disease management. 2015;3-23. https://doi.org/10.1007/978-3-319-23075-7_1Eberlein C, Heuer H, Vidal S, Westphal A. Microbial communities in Globodera pallida females raised in potato monoculture soil. Phytopathology. 2016;106(6):581-90. https://doi.org/10.1094/PHYTO-07-15-0180-R, PMid:26863445Gao X, Yin B, Borneman J, Becker JO. Assessment of parasitic activity of Fusarium strains obtained from a Heterodera schachtii-suppressive soil. J Nematol. 2008;40(1):1-6. PMID: 19259511Shi W, Li M, Wei G, Tian R, Li C, Wang B, et al. The occurrence of potato common scab correlates with the community composition and function of the geocaulosphere soil microbiome. Microbiome. 2019;7(1):1-18. https://doi.org/10.1186/s40168-019-0629-2Mendes R, Garbeva P, Raaijmakers JM. The rhizosphere microbiome: Significance of plant beneficial, plant pathogenic, and human pathogenic microorganisms. FEMS Microbiol Rev. 2013;37(5):634-63. https://doi.org/10.1111/1574-6976.12028, PMid: 23790204Raaijmakers JM, Mazzola M. Diversity and natural functions of antibiotics produced by beneficial and plant pathogenic bacteria. Annu Rev Phytopathol. 2012;50:403-24. https://doi.org/10.1146/annurev-phyto-081211-172908, PMid:22681451Vinale F, Sivasithamparam K, Ghisalberti EL, Woo SL, Nigro M, Marra R, et al. Trichoderma secondary metabolites active on plants and fungal pathogens. Open Mycol J. 2014;8(1). https://doi.org/10.2174/1874437001408010127EPPO (European and Mediterranean Plant Protection Organization). PM 7/40(4) Globodera rostochiensis and Globodera pallida. Bull OEPP/EPPO Bull. 2017;47:174-197. https://doi.org/10.1111/epp.12391Kachiprath B, Jayanath G, Solomon S, Sarasan M. CTAB influenced differential elution of metagenomic DNA from saltpan and marine sediments. 3 Biotech. 2018 Jan;8:1-5. https://doi.org/10.1007/s13205-017-1078-x, PMid:29354355 PMCid:PMC5752666Andrews S. FastQC: a quality control tool for high throughput sequence data [Internet]. 2010. Available from: https://www.bioinformatics.babraham.ac.uk/projects/fastqcSchmieder R, Edwards R. Quality control and preprocessing of metagenomic datasets. Bioinformatics. 2011;27(6):863-864. https://doi.org/10.1093/bioinformatics/btr026Blanco-Míguez A, Beghini F, Cumbo F, McIver LJ, Thompson KN, Zolfo M, et al. Extending and improving metagenomic taxonomic profiling with uncharacterized species using MetaPhlAn 4. Nat Biotechnol. 2023;41(11):1633-1644. https://doi.org/10.1038/s41587-023-01688-w, PMid:36823356 PMCid:PMC10635831Tamames J, Puente-Sánchez F. SqueezeMeta, a highly portable, fully automatic metagenomic analysis pipeline. Front Microbiol. 2019;9:3349. https://doi.org/10.3389/fmicb.2018.03349, PMid:30733714 PMCid:PMC6353838Li D, Liu CM, Luo R, Sadakane K, Lam TW. MEGAHIT: an ultra-fast single-node solution for large and complex metagenomics assembly via succinct de Bruijn graph. Bioinformatics. 2015;31(10):1674-1676. https://doi.org/10.1093/bioinformatics/btv033, PMid:25609793Wang Q, Garrity GM, Tiedje JM, Cole JR. Naive Bayesian classifier for rapid assignment of rRNA sequences into the new bacterial taxonomy. Appl Environ Microbiol. 2007;73(16):5261-5267. https://doi.org/10.1128/AEM.00062-07Hyatt D, Chen GL, LoCascio PF, Land ML, Larimer FW, Hauser LJ. Prodigal: prokaryotic gene recognition and translation initiation site identification. BMC Bioinformatics. 2010;11:1-11. https://doi.org/10.1186/1471-2105-11-119, PMid:20211023 PMCid:PMC2848648Buchfink B, Xie C, Huson DH. Fast and sensitive protein alignment using DIAMOND. Nat Methods. 2015;12(1):59-60. https://doi.org/10.1038/nmeth.3176, PMid:25402007Eddy SR. A new generation of homology search tools based on probabilistic inference. Genome Inform. 2009;23(1):205-211. https://doi.org/10.1142/9781848165632_0019Finn RD, Coggill P, Eberhardt RY, Eddy SR, Mistry J, Mitchell AL, et al. The Pfam protein families' database: towards a more sustainable future. Nucleic Acids Res. 2016; 44(D1):D279-D285. https://doi.org/10.1093/nar/gkv1344, PMid:26673716 PMCid:PMC4702930Joseph TA, Pe'er I. An Introduction to Whole-Metagenome Shotgun Sequencing Studies. 2021;107-22. https://doi.org/10.1007/978-1-0716-1103-6_6, PMid:33606255Masuda Y, Mise K, Xu Z, Zhang Z, Shiratori Y, Senoo K, et al. Global soil metagenomics reveals distribution and predominance of Deltaproteobacteria in nitrogen-fixing microbiome. Microbiome. 2024 May 24;12(1):95. https://doi.org/10.1186/s40168-024-01812-1Zheng Y, Gong X. Niche differentiation rather than biogeography shapes the diversity and composition of microbiome of Cycas panzhihuaensis. Microbiome 2019;7:152. https://doi.org/10.1186/s40168-019-0770-yPalleroni NJ. Introduction to the Family Pseudomonadaceae. The Prokaryotes. Berlin, Heidelberg: Springer Berlin Heidelberg, 1981;655-65. https://doi.org/10.1007/978-3-662-13187-9_58Deka B, Baruah C, Babu A. Entomopathogenic microorganisms: their role in insect pest management. Egypt J Biol Pest Control 2021;31:121. https://doi.org/10.1186/s41938-021-00466-7Saxena AK, Kumar M, Chakdar H, Anuroopa N, Bagyaraj DJ. Bacillus species in soil as a natural resource for plant health and nutrition. Journal of applied microbiology. 2020 Jun 1;128(6):1583-94. https://doi.org/10.1111/jam.14506, PMid:31705597Patani A, Patel M, Islam S, Yadav VK, Prajapati D, Yadav AN, et al. Recent advances in Bacillus-mediated plant growth enhancement: a paradigm shift in redefining crop resilience. World Journal of Microbiology and Biotechnology. 2024 Feb;40(2):77. https://doi.org/10.1007/s11274-024-03903-5, PMid:38253986Berendsen RL, van Verk MC, Stringlis IA, Zamioudis C, Tommassen J, Pieterse CM, et al. Unearthing the genomes of plant-beneficial Pseudomonas model strains WCS358, WCS374 and WCS417. BMC genomics. 2015 Dec;16:1-23. https://doi.org/10.1186/s12864-015-1632-zFlury P, Aellen N, Ruffner B, Péchy-Tarr M, Fataar S, Metla Z, et al. Insect pathogenicity in plant-beneficial pseudomonads: phylogenetic distribution and comparative genomics. The ISME journal. 2016 Oct;10(10):2527-42. https://doi.org/10.1038/ismej.2016.5Weller DM, Raaijmakers JM, Gardener BBM, Thomashow LS. Microbial populations responsible for specific soil suppressiveness to plant pathogens. Annu Rev Phytopathol. 2002;40(1):309-48. https://doi.org/10.1146/annurev.phyto.40.030402.110010Jiao S, Chen W, Wang J, Du N, Li Q, Wei G. Soil microbiomes with distinct assemblies through vertical soil profiles drive the cycling of multiple nutrients in reforested ecosystems. Microbiome. 2018 Dec;6:1-3. https://doi.org/10.1186/s40168-018-0526-0Dubey A, Malla MA, Khan F, Chowdhary K, Yadav S, Kumar A, et al. Soil microbiome: a key player for conservation of soil health under changing climate. Biodiversity and Conservation. 2019 Jul 30;28:2405-29. https://doi.org/10.1007/s10531-019-01760-5Suman J, Rakshit A, Ogireddy SD, Singh S, Gupta C, Chandrakala J. Microbiome as a key player in sustainable agriculture and human health. Frontiers in Soil Science. 2022 Apr 11;2:821589. https://doi.org/10.3389/fsoil.2022.821589Eze, Chijioke K, Obasi, Patrick N, Ewa, Chikaodis S, et al. Soil Microbiome in Nutrient Conservation for Plant Growth. InProspects for Soil Regeneration and Its Impact on Environmental Protection 2024 Feb 27 (pp. 335-350). Cham: Springer Nature Switzerland. https://doi.org/10.1007/978-3-031-53270-2_15Philippot L, Griffiths BS, Langenheder S. Microbial Community Resilience across Ecosystems and Multiple Disturbances. Microbiol Mol Biol Rev 2021;85. https://doi.org/10.1128/MMBR.00026-20, PMid:33789927 PMCid:PMC8139522Wagg C, Hautier Y, Pellkofer S, Banerjee S, Schmid B, van der Heijden MG. Diversity and asynchrony in soil microbial communities stabilizes ecosystem functioning. elife. 2021 Mar 23;10:e62813. https://doi.org/10.7554/eLife.62813Bossio DA, Girvan MS, Verchot L, Bullimore J, Borelli T, Albrecht A, et al. Soil microbial community response to land use change in an agricultural landscape of western Kenya. Microbial ecology. 2005 Jan;49:50-62. https://doi.org/10.1007/s00248-003-0209-6Cowan DA, Lebre PH, Amon CE, Becker RW, Boga HI, Boulangé A, et al. Biogeographical survey of soil microbiomes across sub-Saharan Africa: structure, drivers, and predicted climate-driven changes. Microbiome. 2022 Aug 23;10(1):131. https://doi.org/10.1186/s40168-022-01297-w, PMid:35996183 PMCid:PMC9396824Hou D, Huang Z, Zeng S, Liu J, Wei D, Deng X, et al. Environmental factors shape water microbial community structure and function in shrimp cultural enclosure ecosystems. Frontiers in microbiology. 2017 Nov 29;8:2359. https://doi.org/10.3389/fmicb.2017.02359Li Y, Wang J, Li E, Yang X, Yang J. Shifts in microbial community structure and Co-occurrence network along a wide soil salinity gradient. Microorganisms. 2024 Jun 22;12(7):1268. https://doi.org/10.3390/microorganisms12071268Yang C, Li K, Lv D, Jiang S, Sun J, Lin H, et al. Inconsistent response of bacterial phyla diversity and abundance to soil salinity in a Chinese delta. Scientific Reports. 2021 Jun 18;11(1):12870. https://doi.org/10.1038/s41598-021-92502-7Fan S, Qin J, Sun H, Jia Z, Chen Y. Alpine soil microbial community structure and diversity are largely influenced by moisture content in the Zoige wetland. International Journal of Environmental Science and Technology. 2022 May;19(5):4369-78. https://doi.org/10.1007/s13762-021-03287-1Wang Z, Bai Y, Hou J, Li F, Li X, Cao R, et al. The changes in soil microbial communities across a subalpine forest successional series. Forests. 2022 Feb 11;13(2):289. https://doi.org/10.3390/f13020289Shade A, Peter H, Allison SD, Baho DL, Berga M, Bürgmann H, et al. Fundamentals of microbial community resistance and resilience. Front Microbiol. 2012;3:417. https://doi.org/10.3389/fmicb.2012.00417Ali A, Imran Ghani M, Li Y, Ding H, Meng H, Cheng Z. Hiseq base molecular characterization of soil microbial community, diversity structure, and predictive functional profiling in continuous cucumber planted soil affected by diverse cropping systems in an intensive greenhouse region of northern China. International Journal of Molecular Sciences. 2019 May 28;20(11):2619. https://doi.org/10.3390/ijms20112619Xie ZX, Yan KQ, Kong LF, Gai YB, Jin T, He YB, et al. Metabolic tuning of a stable microbial community in the surface oligotrophic Indian Ocean revealed by integrated meta-omics. Marine Life Science & Technology. 2022 May;4(2):277-90. https://doi.org/10.1007/s42995-021-00119-6, PMid:37073226 PMCid:PMC10077294McCarty GW. The role of glutamine synthetase in regulation of nitrogen metabolism within the soil microbial community. The Significance and Regulation of Soil Biodiversity. Dordrecht: Springer Netherlands, 1995;153-9. https://doi.org/10.1007/978-94-011-0479-1_13Cai H, Zhou Y, Xiao J, Li X, Zhang Q, Lian X. Overexpressed glutamine synthetase gene modifies nitrogen metabolism and abiotic stress responses in rice. Plant cell reports. 2009 Mar;28:527-37. https://doi.org/10.1007/s00299-008-0665-z, PMid:19123004Alves JA, Leal FC, Previato-Mello M, da Silva Neto JF. A quorum sensing-regulated type VI secretion system containing multiple nonredundant VgrG proteins is required for interbacterial competition in Chromobacterium violaceum. Microbiology Spectrum. 2022 Aug 31;10(4):e01576-22. https://doi.org/10.1128/spectrum.01576-22Liang X, Zheng HY, Zhao YJ, Zhang YQ, Pei TT, Cui Y, et al. VgrG Spike Dictates PAAR Requirement for the assembly of the type VI secretion system. Journal of Bacteriology. 2023 Feb 22;205(2):e00356-22. https://doi.org/10.1128/jb.00356-22Wilpiszeski RL, Aufrecht JA, Retterer ST, Sullivan MB, Graham DE, Pierce EM, et al. Soil aggregate microbial communities: towards understanding microbiome interactions at biologically relevant scales. Applied and environmental microbiology. 2019 Jul 15;85(14):e00324-19. https://doi.org/10.1128/AEM.00324-19de Sousa LP, Guerreiro-Filho O, Mondego JMC. The Rhizosphere Microbiomes of Five Species of Coffee Trees. Burbank LP (ed.). Microbiol Spectr 2022;10, DOI: 10.1128/spectrum.00444-22. https://doi.org/10.1128/spectrum.00444-22Aravind L, Anantharaman V, Balaji S, Babu MM, Iyer LM. The many faces of the helix-turn-helix domain: transcription regulation and beyond. FEMS microbiology reviews. 2005 Apr 1;29(2):231-62. https://doi.org/10.1016/j.femsre.2004.12.008, PMid:15808743Knapp GS, Hu JC. Specificity of the E. coli LysR-Type Transcriptional Regulators. Herman C (ed.). PLoS One 2010;5: e15189. https://doi.org/10.1371/journal.pone.0015189Moummou H, Kallberg Y, Tonfack LB, Persson B, van Der Rest B. The plant short-chain dehydrogenase (SDR) superfamily: genome-wide inventory and diversification patterns. BMC plant biology. 2012 Dec;12:1-7. https://doi.org/10.1186/1471-2229-12-219Nochi Z, Olsen RKJ, Gregersen N. Short-chain acyl-CoA dehydrogenase deficiency: from gene to cell pathology and possible disease mechanisms. J Inherit Metab Dis 2017;40:641-55. https://doi.org/10.1007/s10545-017-0047-1, PMid:28516284Cong J, Yang Y, Liu X, Lu H, Liu X, Zhou J, et al. Analyses of soil microbial community compositions and functional genes reveal potential consequences of natural forest succession. Scientific reports. 2015 May 6;5(1):10007. https://doi.org/10.1038/srep10007Feng G, Xie T, Wang X, Bai J, Tang L, Zhao H, et al. Metagenomic analysis of microbial community and function involved in cd-contaminated soil. BMC microbiology. 2018 Dec;18:1-3. https://doi.org/10.1186/s12866-018-1152-5Ma X, Song Y, Song C, Wang X, Wang N, Gao S, et al. Effect of nitrogen addition on soil microbial functional gene abundance and community diversity in permafrost peatland. Microorganisms. 2021 Dec 2;9(12):2498. https://doi.org/10.3390/microorganisms9122498Singavarapu B, Du J, Beugnon R, Cesarz S, Eisenhauer N, Xue K, et al. Functional potential of soil microbial communities and their subcommunities varies with tree mycorrhizal type and tree diversity. Microbiology Spectrum. 2023 Apr 13;11(2):e04578-22. https://doi.org/10.1128/spectrum.04578-22, PMid:36951585 PMCid:PMC10111882Bhardwaj D, Ansari MW, Sahoo RK, Tuteja N. Biofertilizers function as key player in sustainable agriculture by improving soil fertility, plant tolerance and crop productivity. Microbial cell factories. 2014 Dec;13:1-0. https://doi.org/10.1186/1475-2859-13-66Pattnaik BK, Sahu C, Choudhury S, Santra SC, Moulick D. Importance of soil management in sustainable agriculture. InClimate-Resilient Agriculture, Vol 1: Crop Responses and Agroecological Perspectives 2023 Nov 10 (pp. 487-511). Cham: Springer International Publishing. https://doi.org/10.1007/978-3-031-37424-1_22Chai Y, Cao Y, Yue M, Tian T, Yin Q, Dang H, et al. Soil abiotic properties and plant functional traits mediate associations between soil microbial and plant communities during a secondary forest succession on the Loess Plateau. Frontiers in Microbiology. 2019 Apr 26;10:895. https://doi.org/10.3389/fmicb.2019.00895, PMid:31105679 PMCid:PMC6499021Lehmann J, Bossio DA, Kögel-Knabner I, Rillig MC. The concept and future prospects of soil health. Nature Reviews Earth & Environment. 2020 Oct;1(10):544-53. https://doi.org/10.1038/s43017-020-0080-8, PMid:33015639 PMCid:PMC7116140Anthony WE, Allison SD, Broderick CM, Chavez Rodriguez L, Clum A, Cross H, et al. From soil to sequence: filling the critical gap in genome-resolved metagenomics is essential to the future of soil microbial ecology. Environmental Microbiome. 2024 Aug 2;19(1):56. https://doi.org/10.1186/s40793-024-00599-w, PMid:39095861 PMCid:PMC11295382
